# Prevalence of vitamin D deficiency across the spectrum of glucose intolerance

**DOI:** 10.1186/s40200-015-0179-5

**Published:** 2015-06-30

**Authors:** K. D. Modi, Md Ishaq Ahmed, Rajesh Chandwani, K. V. S. Hari Kumar

**Affiliations:** Department of Endocrinology, Dr Modi’s Clinic, Mehdipatnam, Hyderabad, India; Department of Personnel & Industrial Relations, IIM, Ahmedabad, India; Department of Endocrinology, Command Hospital, Chandimandir, Haryana India

**Keywords:** Vitamin D, Type 2 Diabetes, Prevalence, Prediabetes, India

## Abstract

**Background:**

Vitamin D deficiency (VDD) is inversely associated with insulin resistance. We studied the prevalence of VDD across the spectrum of glucose intolerance, including normal glucose tolerance (NGT), prediabetes (PD) and type 2 diabetes (T2D).

**Methods:**

We conducted this cross-sectional, observational study by serially including the PD and T2D patients seen between June and December 2014. We excluded patients with major illness, secondary diabetes and use of vitamin D or glucocorticoids. VDD was defined as serum 25-hydroxy vitamin D (25OHD) less than 30 ng/mL. The study population was divided into 3 groups: T2D (Group 1; n = 274), PD (Group 2; n = 62) and NGT controls (Group 3; n = 270) for the analysis and appropriate statistical methods were used.

**Results:**

The study participants (n = 606, 28 % males) had a mean age of 43.2 ± 13.6 years, BMI of 27.7 ± 5.9 kg/m^2^, HbA1c of 6.6 ± 2 % and mean 25OHD of 18.8 ± 15.7 ng/mL. VDD was seen in 85 % of the entire study population including 84 % in T2D, 77 % in prediabetes and in 87 % of the controls. The mean 25OHD levels were lower in the control group (16.8 ng/mL) when compared with T2D and prediabetes (19.9 and 22.4 ng/mL) respectively (P = 0.0124). Univariate analysis showed higher odds of VDD in females (P < 0.0001) but no association with diabetes, age, BMI and HbA1c.

**Conclusion:**

Our data showed that VDD is prevalent in the majority of the population, irrespective of the underlying glucose intolerance. Further studies are required to determine the association between the vitamin D and diabetes.

## Background

Vitamin D deficiency (VDD) is the most widely reported nutritive deficiency leading to increased concern amongst the general public and medical fraternity [1]. The skeletal role of vitamin D was established in the last century and vitamin D therapy leads to a marked reduction in the prevalence of rickets and osteomalacia [2]. Researchers have identified the presence of vitamin D receptors in many extra skeletal tissues, giving an expanded role for vitamin D. The extensive actions of vitamin D prompted few investigators to label the compound as a hormone instead of vitamin [3]. This deficiency has been implicated as a risk factor in many autoimmune, metabolic and neoplastic disorders during the last decade [4]. Observational studies have suggested that VDD has been associated with a myriad of metabolic abnormalities, including hypertension, diabetes, dyslipidemia and obesity [5]. Previous reports suggest the presence of VDD in more than 90 % of Indians and the neighboring Asian countries, whereas it falls to about 40 % in patients from the developed countries [6].

The role of vitamin D has been established in the carbohydrate metabolism, insulin secretion and its intracellular action [7]. During the last decade, there is an exponential rise in the research pertaining to vitamin D in glucose intolerance. Epidemiological studies suggest a higher risk of type 2 diabetes (T2D) in patients with VDD and studies are exploring the therapeutic role of vitamin D in the management of diabetes [8]. T2D and prediabetes (PD) are a major public health problem in India with a population prevalence of 13 % together [9]. Prediabetes is a harbinger of the future T2D with an approximate conversion rate of 5 % annually [10]. The prevalence of VDD has not been studied across the spectrum of diabetes in India and very few reports exist from other developed countries [11]. Hence, we conducted this study to assess the prevalence of VDD in patients with PD and T2D.

## Methods

### Study population

This was a cross-sectional, observational study conducted at two tertiary level hospitals located in south India (latitude: 17^0^ 37^′^) and north India (latitude: 30^0^ 74^′^). The participants were recruited from the outpatient department of the hospital. Serial patients with a diagnosis of T2D and PD, aged between 18 and 65 years seen between June 2014 and December 2014 were included in the study. We excluded patients with any major illness (end stage kidney disease, cirrhosis, pancreatitis, cancer and cardiovascular disorders), recent surgery or diabetic ketoacidosis in last 6 months, use of glucocorticoids or vitamin D and secondary diabetes. Control population was derived from the patients attending the obesity and thyroid clinic with normal glucose tolerance (NGT) and are free from any systemic illness. We included equal number of controls to the T2D patients (within 5 % variation to minimise the statistical error in final analysis) and all the control population had normal thyroid and glucose profile on biochemical measurements. The patients were divided into 3 groups for the analysis as per the underlying diagnosis: Group 1 (T2D; n = 274), Group 2 (PD; n = 62) and Group 3 (NGT; n = 270).

### Study measures

Clinical data and demographic details like age, sex, family history of diabetes and duration of diabetes were obtained from all the participants through personal interview method. The study population hails from different regions of our country with varying dietary patterns. Hence, we did not include the dietary patterns influencing the vitamin D in the study questionnaire. Weight was recorded on a digital weighing scale using OMRON HN 286 (Omron Corporation, Kyoto, Japan with a sensitivity of 100 g), height by using a SWWS05 stadiometer (Multicare Company, Delhi, India with a sensitivity of 0.1 cm) and body mass index (BMI) was calculated as weight in kilograms divided height in meters squared [12]. The blood pressure was checked by using an OMRON-HEM 7120 electronic instrument over right arm in the sitting position, and three readings were taken at one minute intervals. The average of all three measures was taken for the study purpose. Fasting venous blood samples were collected after an overnight fast for more than 8 h and analyzed for glucose, glycosylated hemoglobin (HbA1c) and 25-hydroxy vitamin D (25OHD). Post meal blood sample for glucose was collected 2 h after meal consumption. Plasma glucose was estimated by the glucose oxidase method, HbA1c was estimated by high pressure liquid chromatography method and 25OHD by the chemiluminescence method. The coefficients of variation for HbA1c, glucose, and 25OHD were 8, 10, and 7.5 %, respectively at our laboratory. The local ethics committee (Independent Review Board, Dr Modi's Clinic and Institutional Ethical Committee, Command Hospital) approved the study protocol at both the centres and all patients provided written informed consent. We followed the international ethical guidelines issued by the World Health Organization to conduct this study [13].

### Definitions

Glucose intolerance is defined as the presence of either prediabetes (impaired fasting glucose or impaired glucose tolerance) or T2D. We used the American Diabetes Association (ADA) recommendations for the diagnosis of prediabetes and T2D [14]. Endocrine society clinical practice guidelines define vitamin D deficiency as levels of 25OHD < 20 ng/mL, vitamin D insufficiency as 25OHD between 21 and 29 ng/mL and normal status as 25OHD level more than 30 ng/mL [15]. In our study, we considered any value including and above 30 ng/mL as normal and less than 30 ng/mL as deficiency for the sake of clarity and analysis. All the participants with hypovitaminosis D were given oral replacement of vitamin D after collection of the blood sample.

### Statistical analysis

Data are presented as mean, standard deviation (SD) and descriptive statistics were used for the data analysis. Sprearman’s correlation and Kruskal-Wallis test (post hoc analysis with Bonferroni method) was used to compare between the data between the groups. We used the nonparametric methods for comparison because the sample was derived from a specialized group attending the hospital and not derived from the population. To identify the associations with vitamin D, univariate logistic regression analysis was conducted with vitamin D deficiency (Yes/No) as a dependent variable and others as independent continuous variables. The results are expressed as odds ratio (OR) and 95 % confidence intervals (CI). A two-tailed p value of less than 0.05 was considered significant for all the tests. The statistical analysis and graph generation was done using the Graph Pad Prism Software, Version 6 (Graph Pad Software, San Deigo, CA, USA).

## Results

The study participants (n = 606, 28 % males) had a mean age of 43.2 ± 13.6 years, BMI of 27.7 ± 5.9 kg/m^2^, HbA1c of 6.6 ± 2 % and mean 25OHD of 18.8 ± 15.7 ng/mL. VDD was seen in 84 % of T2D, 77 % of prediabetes and 87 % of the control population (P = 0.9637). In the entire sample only 90 out of 606 (15 %) patients had normal vitamin D levels. The baseline parameters and detailed comparison between the three groups are given in Table 1. The mean 25OHD levels were lower in the control group when compared with T2D and prediabetes (16.8, 19.9, 22.4 ng/mL, P = 0.0124) patients. Briefly, the results show that the patients with glycemic intolerance were older and females were in the majority in all the three groups. Systolic and diastolic blood pressures were higher in the diabetes population and the BMI was comparable between the three groups. *Post hoc* analysis revealed that 25OHD was significantly less in Group 3 compared to Group 1 and the same was significantly lower than the Group 2. The analysis also showed a significant difference between Groups 1 and 2 pertaining to all the study parameters except for the blood pressure, BMI and 25OHD values.

**Table 1:** Demographic and biochemical parameters in the three study groups

Feature	Unit	Group 1	Group 2	Group 3	*P* value
		(T2D)	(Prediabetes)	(Controls)	
		n = 274	n = 62	n = 270	
Age	years	50 ± 11.6	42.8 ± 12.8	36.3 ± 12.2	**<0.0001**
Gender	M/F	102/172	15/47	52/218	**<0.0001**
Duration of disease	years	5.9 ± 3.4	*6.1 ± 3.6	-	-
Body Mass Index	kg/m^2^	27.8 ± 5.8	28.5 ± 7.7	27.4 ± 5.6	0.3982
Systolic BP	mm Hg	124.1 ± 15.2	123.5 ± 11.7	116.9 ± 9.1	**<0.0001**
Diastolic BP	mm Hg	79.3 ± 9.2	78.4 ± 9.3	73.4 ± 8.1	**<0.0001**
Fasting glucose	mg/dl	166.7 ± 65.5	103.6 ± 10.8	81.6 ± 11.2	**<0.0001**
Post meal glucose	mg/dl	243.5 ± 93.3	140.2 ± 27.3	118.9 ± 11.4	**<0.0001**
HbA1c	%	8.4 ± 1.6	5.9 ± 0.26	4.9 ± 0.45	**<0.0001**
25-OH Vitamin D	ng/ml	19.9 ± 18.3	22.4 ± 18.6	16.8 ± 11.5	**0.0124**

Mean (SD)

*months

Significant values are highlighted in bold

**Fig. 1:**
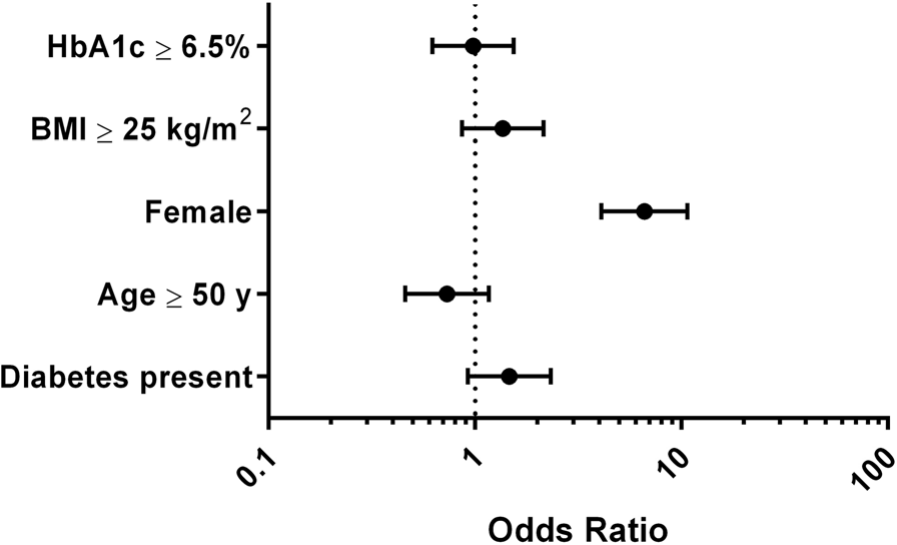
Odds ratio of the study parameters with vitamin D deficiency

Logistic regression models were used to quantify the association between vitamin D levels and the presence of diabetes. The presence of VDD did not differ with the diagnosis of diabetes (OR: 1.4673, CI: 0.9241 – 2.3298), age (OR: 0.7316, CI: 0.4589 – 1.1663), BMI (OR: 1.3641, CI: 0.8648 – 2.1518) and HbA1c (OR: 0.9797, CI: 0.6235 – 1.5394). In view of the lack of association between VDD and diagnosis of diabetes, we did not perform the multivariate analysis. Female sex had higher odds of having a vitamin D deficiency (OR: 6.6132, CI: 4.0922 – 10.6871, P < 0.0001) as shown in Fig. 1. Spearman’s test did not show any correlation between vitamin D level and the age, duration of diabetes and HbA1c (Data not shown).

## Discussion

Our study showed that only 15 % of the study population have normal 25OHD levels. The interesting observation of our study is the higher levels of 25OHD in the glycemic intolerance group than the control population. Similar finding was observed in a recently published report from the neighboring Asian country [11]. Patients with T2D and prediabetes are more exposed to the mass media campaigns, public health awareness programs and also consult the physician frequently for the primary diagnosis [16, 17]. Nevertheless, this could have led to their conscious efforts by increasing the dietary intake of vitamin D coupled with adequate sun exposure to achieve a higher 25OHD level. Population studies from India showed a prevalence of vitamin D in more than 90 % of the patients [18]. Previous reports from other Asian nations also reported VDD in more than 80 % of their population [6, 19]. Our results also suggest that a majority (85 %) of the study population have vitamin D deficiency irrespective of the underlying glycemic status.

Other finding of our study is a markedly higher prevalence of VDD in female sex (Fig. 1). The majority of our clientele hails from Muslim community and their cultural etiquette requires wearing a long robe called “Burqa” by females in outdoors. This additional layer of clothes covers the skin completely preventing the synthesis of vitamin D in the skin [20]. The unique factors responsible for the high prevalence of VDD in our country include dark skin, lack of sunlight exposure, poor dietary consumption and lack of food fortification [21]. The identification of VDD as a modifiable risk factor for skeletal and metabolic disorders prompted the developing countries to fortify the food substances and milk with vitamin D [22].

Multiple mechanisms link the presence of VDD with glycemic intolerance. They include reduction in the calcium mediated insulin secretion, decreased expression of the insulin receptors, increase in the oxidative stress and inflammatory cytokines and low adiponectin levels [4]. Our study showed a trend of higher odds of glycemic intolerance in patients with vitamin D deficiency. However, it was not statistically significant and 25OHD status showed no association with age, obesity and HbA1c. Recent reports suggest that vitamin D is a modifiable risk factor for the obesity and the obesity inducing effects of the FTO gene (name derived from the Fused Toes and Other abnormalities observed in the mice with the specific gene deletion) were observed more in patients with VDD [23, 24]. However, our data did not show any association between the BMI and VDD (OR: 1.3641, CI: 0.8648 – 2.1518). The strength of our study lies in the inclusion of patients from north and south India giving a pan Indian representation, and the findings may be extrapolated to the entire country. The limitations include small sample size, inability to nullify the confounding factors and the cross sectional design of the study. Prospective studies with large number of patients including a representative Indian sample are required to study the link between vitamin D deficiency and other factors related to the diabetes mellitus.

## Conclusion

To conclude, our data showed a high prevalence of VDD in the sample irrespective of the glycemic status. Our study gives more impetus to researchers and policy makers to improve vitamin D status of the population by public education about sun exposure and food fortification to reduce the morbidity associated with VDD.
